# Resilience as a psychiatric factor affecting outcomes after total joint arthroplasty: a systematic review

**DOI:** 10.1186/s42836-024-00240-8

**Published:** 2024-04-05

**Authors:** Andrew G. Kim, Parshva Sanghvi, Adam A. Rizk, Aaron Ahn, Thomas J. Pumo, Atul F. Kamath

**Affiliations:** Department of Orthopaedic Surgery, Cleveland Clinic Foundation, Cleveland, OH 44195 USA

**Keywords:** THA, TKA, Arthroplasty, Resilience, Psychological resilience

## Abstract

**Introduction:**

Mental and psychiatric status continue to be underscreened prior to total joint arthroplasty (TJA). Research on the role of resilience as a psychiatric factor affecting TJA outcomes remains limited. Therefore, our systematic review sought to evaluate the impact of patient resilience in TJA.

**Methods:**

A systematic review of the literature from the Pubmed, MEDLINE, EBSCOhost, and Google Scholar online databases was performed in abidance with Preferred Reporting Items for Systematic Review and Meta-Analyses (PRISMA) guidelines. Studies reporting on outcomes following primary total hip arthroplasty (THA) and/or total knee arthroplasty (TKA) segregated by patient resilience were included. Case reports, reviews, meta-analyses, and conference abstracts were excluded. Primary outcomes of interest included patient-reported outcomes (PROs), surgical outcomes, and postoperative opioid consumption.

**Results:**

Twelve articles were included reporting on a total of 1,577 TJAs. There was a strong agreement that the Patient Reported Outcomes Measurement Information System (PROMIS)-Physical Health and Mental Health components were strongly predicted by patient resilience. However, there was inconclusive evidence regarding the impact of resilience on UCLA Activity Scales (UCLA) and Western Ontario and McMaster Universities Osteoarthritis Index (WOMAC) outcomes as well as postoperative hip and knee function. Similarly, conflicting evidence was presented regarding the effect of resilience on length of stay (LOS). Greater resilience was associated with reduced opioid usage in the immediate inpatient postoperative period. However, resilience had no significant effect on opioid requirements in the postoperative outpatient follow-up time frame.

**Conclusion:**

The present analysis demonstrated mixed, inconclusive evidence regarding the impact of resilience on postoperative outcomes. The paucity of research evaluating this relationship warrants further investigation, examining both short and long-term outcomes. Due to the limited literature evaluating resilience as a predictor of outcomes following TJA, we cannot definitively rule out resilience as a valuable metric and must further examine its utility as a preoperative screening tool.

**Level of evidence:**

III.

## Background

Total joint arthroplasty (TJA) continues to be one of the most common procedures in the United States with over one million total hip and knee replacements performed annually [1]. Numerous modifiable preoperative risk factors have been identified as significant predictors of outcomes following TJA, including obesity, malnutrition, diabetes, and psychiatric conditions [2, 3]. Psychiatric conditions, notably depression and anxiety, have harbored strong interest in recent years with studies observing a strong link between preoperative depression and anxiety status and increased rates of adverse outcomes, such as greater length of stay, readmission, and pain [4]. Despite these new findings, mental and psychiatric status continues to be underscreened prior to TJA procedures.

While depression and anxiety have been gaining traction, research on the role of resilience as a psychiatric factor affecting TJA outcomes remains limited. Resilience is defined as the “ability to adapt and improve when facing adversity or other stressors,” or the ability to bounce back from stress, and is one of many factors that constitute mental health [5]. Though resilience is quantified by various scales, the Connor-Davidson 10-item Resilience Scale (CD-RISC) and the Brief Resilience Scale (BRS) are the most commonly used tools. Higher resilience scores have been linked to positive postoperative patient-reported outcomes (i.e., pain, satisfaction) and earlier return to activity across various orthopaedic procedures, including shoulder, hip, and knee arthroscopy [6–9]. Although recent studies have also begun investigating the role of resilience in TJA procedures [10, 11], to the best of our knowledge, there is currently no comprehensive review regarding the influence of resilience on surgical outcomes. Exploring the predictive value of patient resilience in TJA may better inform providers which patients are at a higher risk for adverse outcomes and would thus require special perioperative care.

Therefore, our systematic review sought to evaluate the impact of patient resilience in TJA, specifically, to answer the question: How is patient resilience associated with patient outcomes in TJA?

## Methods

### Literature search

The Pubmed, MEDLINE, EBSCO Host, and Google Scholar online databases were queried for all studies published between 1 January 1997 to 7 February 2023 that reported on patient resilience in total hip arthroplasty (THA) and/or total knee arthroplasty (TKA). The following MeSH and keywords were employed to search the literature in combination with “AND” and “OR” Boolean operators: “Arthroplasty, Replacement, Hip [MeSH]”; “total hip arthroplasty”; “total hip replacement”; “THA”; “total knee replacement [MeSH]”; “total knee arthroplasty”; “TKA”; “total joint arthroplasty”; “TJA”; “resilience”; “psychological resilience [MeSH]”; “resiliency”. For the purposes of this study, we defined TJA as comprising THA and TKA only.

### Eligibility criteria

The following criteria were established prior to the query to identify studies for inclusion: (1) English, full texts were available, (2) reported on primary THA or TKA patients, (3) studies reporting on surgical or patient-reported outcomes segregated by patient resilience. Exclusion criteria included: (1) duplicate studies across databases, (2) single or double case reports, (3) systematic reviews or meta-analyses, (4) studies reporting on revision TJA, (5) studies reporting on hemiarthroplasty, (6) studies reporting on hip resurfacing, (7) studies reporting on arthroscopy, (8) studies reporting on minimally invasive surgical techniques, and (9) abstracts and preprint articles.

### Study selection

**Fig. 1:**
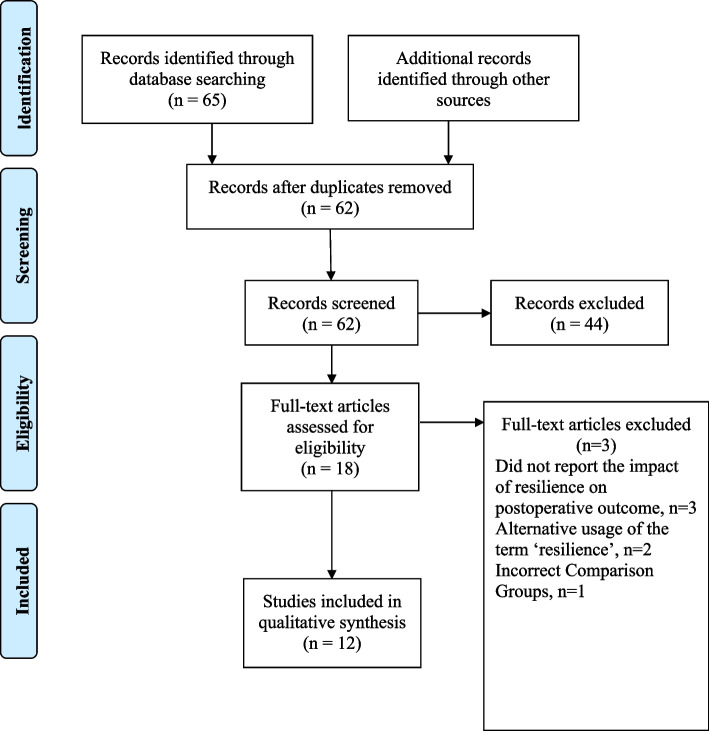
Preferred reporting items for systematic review and meta-analyses (PRISMA) diagram demonstrating article selection process

Two independent reviewers conducted our analysis of the literature in abidance with the Preferred Reporting Items for Systematic Review and Meta-Analyses (PRISMA) guidelines [12]. Disagreements at any stage of the study selection process were evaluated and resolved by a third reviewer. Following the initial query, 62 articles were identified following the exclusion of duplicates. Subsequent evaluation of the titles and abstracts resulted in 18 remaining articles for full-text evaluation. Utilizing the pre-determined inclusion and exclusion criteria, 12 articles were included in the final analysis [10, 11, 13–22]. No additional studies were identified following a stepwise evaluation of each article’s reference list (Fig. 1).

### Methodological quality assessment

The risk of bias was evaluated for all included studies. The Methodological Index for Non-Randomized Studies (MINORS) tool was utilized for all non-randomized studies [23]. The MINORS tool consists of 12 methodological criteria scored between 0 and 2 to total up to an ideal global score of 24. Bias levels are categorized as follows: 0–6 (very low quality), 7–12 (low quality), 13–18 (moderate quality), 19–24 (high quality) [24]. Two independent reviewers applied these tools to assess the risk of bias in each included study. A third reviewer was utilized to resolve any disagreements.

### Included studies

The present analysis included 12 articles reporting on a total of 1,577 TJAs (Table 1). Across the included articles, 4 different metrics for resilience were used, with the BRS (*n* = 7) and CD-RISC (*n* = 3) being the most commonly utilized tools. Due to the heterogeneity of the included studies, a pooled analysis could not be performed. The results of the studies are presented descriptively.

**Table 1:** Included studies

Study	Design	Procedure	RS	Sample Size	MINORS score	Primary Outcomes
Trinh et al., 2021 [10]	Prospective	THA, TKA	BRS	98	19	PROMIS-10
Trinh et al., 2022 [13]	Retrospective	THA, TKA	BRS	119	19	Opioid Consumption
Zabat et al., 2022 [14]	Retrospective	THA	BRS	393	19	HOOS, JR
Bumberger et al., 2022 [15]	Prospective	THA	CD-RISC	103	17.5	WOMAC and UCLA score
Magaldi et al., 2019 [11]	Prospective	TKA	BRS	153	21	KOOS, JR and PROMIS-10
Lynskey et al., 2021 [16]	Prospective	THA, TKA	CD-RISC	140	22	Satisfaction VAS and NPS
March et al., 2022 [17]	Prospective	TKA	BRS	75	21	WOMAC and NPRS (Primary outcome was acute hospital LOS)
Bumberger et al., 2021 [18]	Prospective	TKA	CD-RISC	163	20	WOMAC and UCLA score
Nwankwo et al., 2021 [19]	Prospective	TKA	BRS	117	21	KOOS, JR and PROMIS Physical Health/Mental Health
Benditz et al., 2017 [20]	Prospective	THA	RS-11	50	17	HHS
Haffar et al., 2021 [21]	Retrospective	TKA	BRS	86	18.5	KSS, KOOS, JR and VR-12 MCS
Sciume et al., 2018 [22]	Prospective	THA, TKA	RS-10	80	16.5	FIM; WHOQOL-BREF

*THA* Total hip arthroplasty, *TKA* Total knee arthroplasty, *BRS* Brief Resilience Scale, *CD-RISC* Connor-Davidson 10-item Resilience Scale, *RS* Resilience Scale, *MINORS* Methodological Index for Non-Randomized Studies, *PROMIS* Patient-Reported Outcomes Measurement Information System, *HOOS, JR* Hip dysfunction and Osteoarthritis Outcome Score for Joint Replacement, *WOMAC* Western Ontario and McMaster Universities Osteoarthritis Index, *KOOS, JR* Knee injury and Osteoarthritis Outcome Score for Joint Replacement, *VAS* Visual analog scale, *NPS* Net Promoter Score, *LOS* Length of stay, *HHS* Harris Hip Score, *KSS* Knee Society Score, *VR-12 MCS* Veterans Rand 12 Mental Component Score, *FIM* Functional Independence Measure, *WHOQOL-BREF* World Health Organization Quality of Life Brief Version

All 12 of the included articles were classified as non-randomized studies. The average MINORs score for the studies included in our analysis was 19.29 (standard deviation [SD] = 1.75). 3 studies were of moderate quality, and 9 studies were of high quality.

### Primary outcome

The primary goal of the study was to investigate the association between patient resilience and surgical and patient reported outcome measurements (PROMs) (Table 2). All 12 of the included studies reported on the impact of patient resilience on surgical and PROMs [10, 11, 13–22].

**Table 2:** Impact of resilience on outcomes

Study	Key Findings
Trinh et al., 2021 [10]	Higher preoperative BRS scores significantly correlated with greater PROMIS-PH (*r* = 0.49, *P* < 0.001) and MH (*r* = 0.47, *P* < 0.001) outcomes at 1 year follow-up. Furthermore, significant differences in PROMIS-PH (42.3 vs. 47.7 vs. 54.1, *P* < 0.001) and MH (45.8 vs. 50.8 vs. 53.3, *P* < 0.001) scores were observed across low, normal, and high resilience groups 1 year postoperatively. Although not statistically significant, resilience groups additionally differed in the proportion of patients who reached MCID for PROMIS-PH (*P* = 0.056) and MH (*P* = 0.135)
Trinh et al., 2022 [13]	Resilience did not significantly impact the use of opioids in the perioperative period. However, in the immediate inpatient postoperative period, high resilience patients required significantly fewer opioids than patients with low resilience (2.12 MME/h vs. 3.11 MME/h, *P* = 0.035). Higher preoperative BRS scores also strongly correlated with reduced inpatient opioid usage (*r* = -0.026, *P* = 0.003). Interestingly, no significant differences were observed across resilience groups for postoperative outpatient opioid use regarding both initial prescription and total refills
Zabat et al., 2022 [14]	At 3-month follow-up, HOOS JR scores significantly increased from the low to high resilience patient groups (low vs. normal vs. high: 72.5 ± 16.3 vs. 75.9 ± 15.5 vs. 81.0 ± 12.7, *P* = 0.03) despite no baseline differences in HOOS JR scores Increasing resilience was significantly associated with shorter LOS. The average LOS for low, normal, and high resilience groups were 44.29 ± 49.13, 31.14 ± 26.86, and 10.97 ± 15.00 h, respectively. Normal and high-resilience patients were also more likely to be discharged on the same day compared to low-resilience patients (OR: 1.49 and 3.01 respectively, *P* = 0.01)
Bumberger et al., 2022 [15]	WOMAC scores were significantly predicted by resilience (*r* = 0.248, *P* = 0.008), but UCLA scores were not (*r* = 0.045, *P* = 0.332)
Magaldi et al., 2019 [11]	3-month outcomes: The change in *r*^2^ attributable to preoperative resilience for PROMIS-PH and MH scores was 0.06 (*P* = 0.001) and 0.15 (*P* < 0.001), respectively. For EQ-5D, the change in *r*^2^ attributable to preoperative resilience was 0.10 (*P* < 0.001). KOOS JR scores were not significantly impacted by preoperative resilience 3 months postoperatively. The change in *r*^2^ attributable to concurrent resilience was 0.10 (*P* < 0.001) for KOOS JR scores, 0.24 (*P* < 0.001) for EQ-5D scores, 0.18 (*P* < 0.001) for PROMIS-PH scores, and 0.27 (*P* < 0.001) for PROMIS-MH scores 1-year outcomes: The change in *r*^2^ attributable to preoperative resilience for the PROMIS-PH and MH scores was 0.10 (*P* < 0.001) and 0.20 (*P* < 0.001), respectively. The change in *r*^2^ attributable to preoperative resilience for the EQ-5D score was 0.16 (*P* < 0.001). Similar to the 3-month follow-up, KOOS JR scores were not significantly impacted by preoperative resilience. The change in *r*^2^ attributable to concurrent resilience was 0.05 (*P* = 0.006), 0.17 (*P* < 0.001), 0.09 (*P* < 0.001), and 0.26 (*P* < 0.001) for KOOS JR, EQ-5D, PROMIS-PH, and PROMIS-MH scores respectively
Lynskey et al., 2021 [16]	There was a strong positive correlation between CD-RISC and EQ-VAS (*r* = 0.530, *P* < 0.001), a moderate positive correlation between CD-RISC and Satisfaction VAS (*r* = 0.311, *P* < 0.001), and a moderate positive correlation between CD-RISC and the NPS (*r* = 0.393, *P* < 0.001). Resilient patients (CD-RISC > 60%) had better EQ-VAS (86 vs. 72, *P* < 0.001), Satisfaction VAS (93 vs. 85, *P* = 0.02), and NPS (9.5 vs. 8.4, *P* < 0.001) compared with Less Resilient (CD-RISC < 40%) patients
March et al., 2022 [17]	There was no significant correlation between resilience and LOS (*r* = -0.209, *P* = 0.072). There was no significant difference detected in LOS between groups based on resilience (Mann–Whitney *U* = 330.0, *P* = 0.478). Furthermore, there were no significant differences in inpatient rehabilitation use (Mann–Whitney* U* = 305.50, *P* = 0.188) or physiotherapy inpatient occasions of service (Mann–Whitney *U* = 344.0, *P* = 0.618) between high and low resilience groups
Bumberger et al., 2021 [18]	Upon admission for rehabilitation, WOMAC scores were not significantly predicted by resilience (*r* = 0.127, *P* = 0.118). On the other hand, UCLA scores were significantly predicted by resilience (*r* = 0.257, *P* = 0.001)
Nwankwo et al., 2021 [19]	At the 3-month postoperative timepoint, resilience was positively correlated with KOOS JR (*P* = 0.002) as well as PROMIS-PH (*P* < 0.001) and MH (*P* < 0.001) scores following TKA. Statistical significance was lost for PROMIS-MH following adjustment for covariates
Benditz et al., 2017 [20]	Resilience was not associated with HHS outcomes following THA
Haffar et al., 2021 [21]	No significant correlation was observed between BRS and KOOS JR, KSS Patient Expectation, KSS Patient Satisfaction, and KSS Symptoms scores. While patients with greater resilience tended to have higher KSS Functional Activities scores, this finding was not statistically significant (*r* = 0.215, *P* = 0.062). However, resilience did have a significant positive correlation with postoperative VR-12 MCS reported a minimum of 1 year postoperatively (*r* = 0.428, *P* < 0.001). Nonetheless, no significant correlation was found between BRS scores and change in VR-12 MCS, KOOS JR, or any of the aforementioned KSS parameters from the preoperative to minimum 1-year postoperative time-points There was no correlation between BRS scores and rate of reoperations (*r* = -0.002,* P* = 0.989), postoperative complications (*r* = 0.575, *P* = 0.061), or postoperative opioid consumption (*r* = -0.157, *P* = 0.166)
Sciume et al., 2018 [22]	Greater resilience level was significantly associated with higher functional independence measure (FIM) score, which measures a patient’s ability to carry out daily activities of living on their own. However, this finding only held true for fracture patients, and no difference in FIM score was observed between resilience levels for those who underwent elective surgery

*THA* Total hip arthroplasty, *TKA* Total knee arthroplasty, *BRS* Brief Resilience Scale, *CD-RISC* Connor-Davidson 10-item Resilience Scale, *PROMIS-PH* Patient-Reported Outcomes Measurement Information System Physical Health, *PROMIS-MH* Patient-Reported Outcomes Measurement Information System Mental Health, *MCID* Minimal clinically important difference, *MME* Morphine milligram equivalents, *HOOS, JR* Hip dysfunction and Osteoarthritis Outcome Score for Joint Replacement, *WOMAC* Western Ontario and McMaster Universities Osteoarthritis Index, *KOOS, JR* Knee injury and Osteoarthritis Outcome Score for Joint Replacementm *EQ-5D* EuroQol-5D, *VAS* Visual analog scale, *NPS* Net Promoter Score, *LOS* Length of stay, *HHS* Harris Hip Score, *KSS* Knee Society Score, *VR-12 MCS* Veterans Rand 12 Mental Component Score

## Results

### Effects of resilience on outcomes following TJA

A total of 12 studies explored the relationship between patient resilience and outcomes following TJA [10, 11, 13–22].

### PROMs

The most frequently reported outcome measures were PROMs, with 9 of the 12 studies reporting on the impact of patient resilience on PROMs [10, 11, 14–16, 18–21]. The Physical Health (PH) and Mental Health (MH) components of the Patient Reported Outcomes Measurement Information System (PROMIS) were consistently found to be predicted by resilience [10, 11, 19]. Trinh et al. found that higher preoperative BRS scores significantly correlated with superior PROMIS-PH (*r* = 0.49, *P* < 0.001) and MH (*r* = 0.47, *P* < 0.001) outcomes 1 year postoperatively in TJA [10]. Nwankwo et al. similarly observed a significant association between BRS scores and 3-month PROMIS-PH (*P* < 0.001) and MH (*P* < 0.001) scores in a cohort of TKA patients; however, significance was lost for the PROMIS-MH component following adjustment for covariates [19]. In line with the previous findings, Magaldi et al. found preoperative resilience to significantly predict PROMIS-PH and -MH scores following TKA at both the 3-month and 1-year postoperative time-points [11]. In contrast to the strong agreement regarding the influence of resilience on PROMIS-PH and MH outcomes, there was disagreement on the impact of resilience on the Western Ontario and McMaster Universities Osteoarthritis Index (WOMAC) and UCLA Activity Scale (UCLA) scores [15, 18] following TJA. Amongst TKA patients, Bumberger et al. found that WOMAC scores upon admission for rehabilitation were not predicted by resilience (*r* = -0.127, *P* = 0.118), while the UCLA scores were significantly predicted by resilience (*r* = 0.257, *P* = 0.001) [18]. Conversely, another study conducted by Bumberger et al. examining THA patients had opposing findings with WOMAC scores (*r* = -0.248, *P* = 0.008), but not UCLA scores (*r* = 0.045, *P* = 0.332), being predicted by resilience [15].

Mixed evidence was presented regarding the impact of resilience on postoperative knee function. While Nwankwo et al. found resilience, as measured by the BRS, to be positively correlated with Knee injury and Osteoarthritis Outcome Score (KOOS, JR) scores (*P* = 0.002) 3 months following TKA, Magaldi et al. noted that preoperative BRS scores did not significantly predict KOOS JR. outcomes at both 3 months and 1 year after TKA [11, 19]. Likewise, Haffar et al. observed no correlation between BRS and postoperative KOOS JR scores as well as various Knee Society Score (KSS) parameters postoperatively, including patient expectation, patient satisfaction, functional activities and symptoms scores. Furthermore, there was no correlation between resilience and the change in KOOS JR or any KSS domain from the preoperative to minimum 1-year postoperative time-point [21].

No consensus was reached with regards to the impact of resilience on postoperative hip function. In a study evaluating THA patients, 3-month HOOS JR scores significantly increased from the low to high resilience groups (low vs. normal vs. high: 72.5 ± 16.3 vs. 75.9 ± 15.5 vs. 81.0 ± 12.7; *P* = 0.03) despite no baseline differences in Hip injury and Osteoarthritis Outcome Score (HOOS JR) scores [14]. On the other hand, Benditz et al. found that resilience was not significantly associated with the Harris Hip Score (HHS) outcomes in THA patients [20].

Other notable reported PROMs included the Veterans Rand 12-Item Survey (VR-12) Mental Component Score (MCS), EuroQol (EQ)-5D, and various visual analogue scale (VAS) metrics. No correlation between resilience and improvement in VR-12 MCS was observed [21]. Both the EQ-5D and EQ-VAS were significantly impacted by resilience with Magali et al. showing the BRS to be a significant predictor of 3-month (*P* < 0.001) and 1-year (*P* < 0.001) EQ-5D scores and Lynskey et al. demonstrating a strong positive correlation between CD-RISC and EQ-VAS (*r* = 0.530, *P* < 0.001) [11, 16]. Lynskey et al. further observed a moderate positive correlation between the CD-RISC and Satisfaction VAS (*r* = 0.311, *P* < 0.001) and the CD-RISC and net promoter score (NPS) (*r* = 0.393, *P* < 0.001) [16].

### Surgical outcomes

A total of 4 studies reported surgical and clinician-reported outcomes [14, 17, 21, 22]. Conflicting evidence was presented on the impact of resilience on length of stay (LOS) in TJA. Zabat et al. noted significantly reduced LOS with increasing resilience amongst a cohort of THA patients (*P* < 0.01) [14]. However, in another study examining TKA patients, no significant correlation was found between resilience and LOS (*r* = -0.209, *P* = 0.072), and no differences in LOS were detected between the low resilience (BRS < 3) and normal/high resilience group (BRS ≥ 3) (*P* = 0.478) [17]. Furthermore, no relationship was identified between resilience and rate of reoperations, postoperative complications, and inpatient rehabilitation service use [17, 21]. However, a study by Sciume et al. noted a significant association between greater resilience and a higher functional independence measure (FIM) score, which measures a patient’s ability to carry out daily activities of living on their own. Interestingly, this finding applied only to fracture patients, not those who underwent elective TJA [22].

### Opioid consumption

Opioid consumption was evaluated by a total of 2 articles, which demonstrated mixed results [13, 21]. Trinh et al. reported no significant relationship between resilience and the use of opioids in the perioperative period. However, in the immediate inpatient postoperative period, high-resilience patients had significantly reduced opioid requirements than those with low resilience (high vs. low: 2.12 MME/h vs. 3.11 MME/h, *P* = 0.035, MME = morphine milligram equivalent), and preoperative BRS scores displayed a significant negative correlation with inpatient opioid usage (*r* = -0.026, *P* = 0.003). When evaluating postoperative outpatient opioid requirements at extended follow-up intervals, no differences were observed across resilience groups with regards to both initial prescription and total refills [13]. This was also demonstrated by Haffar et al., who reported no significant correlation between resilience and postoperative opioid usage at a longer follow-up interval of up to 6 months (*r* = -0.157, *P* = 0.166) [21].

## Discussion

The prevalence of psychiatric conditions continues to increase and represents important risk factors for preoperative screening given their association with adverse surgical outcomes for TJA procedures [25, 26]. Previous studies have characterized patient resilience through the CD-RISC and the BRS, demonstrating its associations with improved patient-reported outcomes. In this analysis, we evaluated all studies reporting surgical or patient-reported outcomes by patient resilience during primary THA or TKA procedures. Preoperative resilience significantly predicted increased PROMIS-PH and MH scores in TJA but did not demonstrate a clear association with WOMAC and UCLA scores in addition to postoperative hip and knee function. In a retrospective study evaluating TKA patients, Bumberger et al. observed resilience to significantly predict UCLA, but not WOMAC scores. Prior studies have indicated the UCLA scale as a superior outcome metric for TJA with increased reliability and no floor effects [27]. Therefore, the UCLA scale may better detect differences between low and high resilience patients. However, in another independent study by Bumberger et al. evaluating THA patients, resilience predicted WOMAC, but not UCLA scores. It is important to recognize that these findings were based on a single study for THA and TKA, reinforcing the need to further explore this relationship. Additionally, increased resilience was associated with significantly reduced LOS for THA patients. Finally, there was no consensus on the relationship between resilience and opioid consumption during the postoperative period. This information suggests that further research investigating strategies to increase resilience among patient populations at risk of low resilience may be useful when optimizing TJA during the preoperative, perioperative, and postoperative periods to improve patient and surgical outcomes.

Surgical indications and diagnosis may influence how we interpret patient resilience and utilize it as a risk stratification tool. A prospective study by Sciume et al. found that patients undergoing THA for hip fractures had lower baseline resilience levels than those receiving elective THA [22]. This may be due to a generally lower perceived quality of life and independence in daily activities in those undergoing traumatic surgery compared to their elective counterparts. For traumatic conditions like acute hip fractures, patients abruptly transition from a normal to a dependent state. On the other hand, in degenerative cases like chronic osteoarthritis (OA), patients adapt to their conditions over time. This could partially explain why patients undergoing elective surgery have a higher baseline resilience than hip fracture patients. These findings suggest that resilience may in some way be related to the stressfulness and type of event leading to the surgery (i.e., traumatic vs. elective), although prior studies have suggested that resilience is independent of the type of event [28]. The varying baseline resilience scores across surgical indications and diagnoses should serve to guide expectations regarding patient resilience levels in different cohorts of patients. Additionally, greater resilience levels were significantly associated with higher functional outcomes. Interestingly, this only held true for fracture patients. As for elective surgery, no differences on FIM score existed between high and low levels of resilience [22]. This does not necessarily preclude resilience as an important factor contributing to functional outcome in elective patients. Because elective patients have higher baseline functional scores, it may simply be more challenging to observe significant differences from the preoperative to postoperative period. Conversely, traumatic patients, such as hip fracture patients, tend to have lower baseline functional scores, thereby increasing the probability of observing significant improvements in functional outcomes. Nonetheless, traumatic and elective patients possess distinct clinical characteristics, warranting investigations of the role of resilience in each cohort of patients independently.

There is a paucity of literature evaluating the association between preoperative resilience and LOS following TJA. Our findings revealed mixed evidence on the impact of resilience on LOS, but it is limited by the small number of studies reporting on this relationship. While LOS significantly decreased with increasing resilience amongst THA patients, the same association was not observed amongst TKA patients [14, 17]. This is likely, at least in part, due to the discrepancies in sample size of the studies evaluating THA and TKA. The study evaluating LOS in THA (Zabat et al.) had a sample size of 393, while the study examining TKA (March et al.) had only 75 patients. Therefore, a significant difference is more likely to be observed in THA than TKA. Though the relationship between LOS and resilience is unclear, the potential predictive value of resilience for LOS has important implications. Prolonged LOS can increase risks for inpatient complications and burdensome hospital costs [29]. This could negatively impact both short- and long-term perceived outcomes. While the relationship between LOS and resilience needs further exploration, resilience could be a valuable tool in predicting LOS following TJAs and guiding adequate measures to reduce the deleterious sequelae of increased hospitalization. Additionally, opioid consumption was reported to be higher in low-resilience patients in the immediate inpatient postoperative period. However, no differences in outpatient opioid consumption were observed across resilience levels [13]. This may be related to provider expectations regarding acute inpatient analgesic needs following TJA. For instance, low-resilience patients may receive greater inpatient prescriptions of opioids than high-resilience patients despite comparable postoperative pain levels. There is a need to further investigate postoperative pain outcomes and opioid requirements between resilience levels. Furthermore, if low-resilience patients do have longer inpatient LOS as suggested by Zabat et al. [14], this may further exacerbate the increased inpatient opioid consumption demonstrated in low-resilience patients.

Our analysis has some limitations. There was significant heterogeneity in the resilience metrics utilized as well as the outcomes reported. Therefore, a pooled analysis could not be conducted, and our findings are conveyed descriptively. A pooled analysis was further limited by the relatively sparse literature reporting on the association between resilience and outcomes after TJA. Future endeavors should focus on evaluating previously reported outcomes to improve the consistency and availability of reported data, which will allow for a meta-analysis down the line. Moreover, the wide range of systems used to measure resilience across studies compromises consistency. For example, a patient with a low resilience level according to the BRS may not necessarily have low resilience if measured by another resilience metric. With this in mind, future prospective studies should strive to establish a unified metric for measuring resilience. Based on the included retrospective studies of the present analysis, the BRS is the most commonly utilized metric. Additionally, many of the studies did not control for diagnosed or undiagnosed psychiatric conditions, which may serve as confounding variables that obscure the true association between resilience and postoperative outcomes. While this may be challenging due to many psychiatric conditions being under-diagnosed, studies should attempt to be mindful of such extraneous factors. Most of the included studies did not specify surgical indications or diagnoses, which can influence both resilience and outcomes.

## Conclusion

Our systematic review found inconclusive evidence regarding the impact of preoperative resilience on PROMs and surgical outcomes, including LOS and opioid consumption. Future studies should continue to focus on exploring the relationship between resilience and TJA outcomes as the limited literature makes it challenging to observe any trends or patterns. While the present findings do not support resilience as a significant predictor of outcomes following TJA, the paucity of research on this topic warrants further investigation into whether resilience could be a valuable metric incorporated into routine preoperative screening for TJA to stratify risk and maximize outcomes.

## Data Availability

Not applicable.
